# Pharmacologic activation of peroxisome proliferator-activating receptor-α accelerates hepatic fatty acid oxidation in neonatal pigs

**DOI:** 10.18632/oncotarget.25199

**Published:** 2018-05-08

**Authors:** Kwanseob Shim, Sheila Jacobi, Jack Odle, Xi Lin

**Affiliations:** ^1^ Laboratory of Developmental Nutrition, Department of Animal Sciences, North Carolina State University, Raleigh, NC 27695, USA; ^2^ Current/Present address: Department of Animal Biotechnology, Chonbuk National University, Jeonju, 561-756 Republic of Korea; ^3^ Current/Present address: Ohio Agricultural Research and Development Center, The Ohio State University, Wooster, OH 44691, USA

**Keywords:** pig, liver, PPARα, clofibrate, fatty acid oxidation

## Abstract

Up-regulation of peroxisome proliferator-activating receptor-α (PPARα) and increasing fatty acid oxidation are important for reducing pre-weaning mortality of pigs. We examined the time-dependent regulatory effects of PPARα activation via oral postnatal clofibrate administration (75 mg/(kg-BW·d) for up to 7 days) on mitochondrial and peroxisomal fatty acid oxidation in pigs, a species with limited hepatic fatty acid oxidative capacity due to low ketogenesis. Hepatic oxidation was increased by 44-147% (depending on fatty acid chain-length) and was attained after only 4 days of clofibrate treatment. Acyl-CoA oxidase (ACO) and carnitine palmitoyltransferase I (CPTI) activities accelerated in parallel. The increase in CPTI activity was accompanied by a rapid reduction in the sensitivity of CPTI to malonyl-CoA inhibition. The mRNA abundance of *CPTI* and *ACO,* as well as peroxisomal keto-acyl-CoA thiolase *(KetoACoA)* and mitochondrial malonyl-CoA decarboxylase (*MCD*), also were augmented greatly. However, the increase in ACO activity and *MCD* expression were different from *CPTI,* and significant interactions were observed between postnatal age and clofibrate administration. Furthermore, the expression of acetyl-CoA carboxylase β (*ACCβ*) decreased with postnatal age and clofibrate had no effect on its expression. Collectively these results demonstrate that the expression of PPARα target genes and the increase in fatty acid oxidation induced by clofibrate are time- and age-dependent in the liver of neonatal pigs. Although the induction patterns of *CPTI, MCD, ACO, KetoACoA, and ACCβ* are different during the early postnatal period, 4 days of exposure to clofibrate were sufficient to robustly accelerate fatty acid oxidation.

## INTRODUCTION

Milk fat is a critical macronutrient for the fast growth and development of all mammalian neonates after birth. During the postnatal period of newborn pigs, milk fat becomes the principal substrate for oxidative metabolism, comprising 60% of dietary energy [1]. However, available evidence suggests that the neonatal piglet has a limited capacity to catabolize dietary fatty acids, and a one-day-old pig oxidizes fatty acids at only 32% of the rate of a 24-day-old pig [2]. Metabolic studies with cells have shown that 90% of oleate taken up by piglet hepatocytes is re-esterified with a limited flux through β-oxidation [3]. Moreover, suckling piglets are hypoketonemic despite elevated dietary fat [4, 5]. Therefore, the postnatal regulation of fatty acid oxidation has been studied in newborn piglets to understand milk fat utilization [6–9]. Results indicate that the limited capacity of piglets to utilize fatty acids is in part associated with mutated gene transcription and translation [6–9], as well as the unique protein structures of key enzymes in the fatty acid oxidative pathway [10].

Peroxisome proliferator-activating receptor-α (PPARα) is an essential transcription factor in regulating hepatic fatty acid oxidation. PPARα controls the genes encoding carnitine palmitoyltransferase I (CPTI) [11], mitochondrial 3-hydroxy-3-methyl-glutaryl-CoA synthase (mHMGCS) [12], acyl-CoA oxidase (ACO) [13] and malonyl-CoA decarboxylase (MCD) [14]. The PPARα response element has been identified within the promoters of these genes. Increasing their transcription via PPARα activation increases mitochondrial and peroxisomal fatty acid oxidation [15]. This involves not only the up-regulation of mitochondrial and peroxisomal genes (mainly those involved in β-oxidation), but also an increase in peroxisomal size and the biogenesis of peroxisomes [16]. In mice and rats, PPARα is highly expressed in tissues that have high fatty acid catabolic rates, including the liver, kidney, heart, and skeletal muscle [17]. The critical role of PPARα in controlling fatty acid oxidation and its activation under different physiological adaptation, fasting and refeeding transition, development and aging [18, 19] and pathological conditions such as in alcoholic liver disease [20] and breast cancer [21] have been studied extensively. Contrasted against rodent species, however, PPARα inductive genes for peroxisomal fatty acid oxidation are not highly expressed and peroxisomal proliferation is not sensitive to PPARα activation in weaned pigs [22] and newborn pigs after birth even though milk fat is the primary energy source. The regulatory role of PPARα in fatty acid oxidation in pigs has not been well explored during the neonatal period.

Perinatal programming of metabolism is very dynamic and continues during the early postnatal period. We previously examined the effects of activation of PPARα by clofibrate, the hypolipidemic drug used in human clinical medicine for many years [23], on fatty acid metabolism and milk fat utilization in pigs. As a synthetic PPARα agonist, clofibrate is well known for decreasing circulating lipids, including LDL-cholesterol, and for increasing fatty acid oxidation [24]. Indeed, in concurrent (unpublished) work we confirmed that clofibrate reduced circulating cholesterol (by 33%) and triglycerides (by 26%) while maintaining normoglycemia (110 mg/dL; data not shown) in suckling pigs. We showed that clofibrate effectively stimulated *in vitro* fatty acid oxidation in tissue homogenates and hepatocytes isolated from newborn pigs [25] and *in vivo* oxidation in neonatal pigs following orogastric gavage [26]. Most recently we showed that fatty acid oxidation was increased in newborn pigs after their dams were fed clofibrate during the last week of gestation [27]. The results from our studies demonstrated that PPARα activation indeed played an important role in fatty acid oxidative metabolism in pigs during the fetus-to-newborn transition and suckling periods. However, the kinetics of PPARα induction of fatty acid oxidation was not examined in previous studies. We hypothesized that piglet age and duration of clofibrate administration could be important for optimizing induction of fatty acid oxidation. Furthermore, induction of genes controlling fat oxidation such as *CPTI*, acetyl-CoA carboxylase (*ACC*), and *MCD* has not been examined synchronously as factors affecting oxidation rate.

The work reported herein was designed to better define the time- and age-dependent effects of PPARα activation via postnatal clofibrate administration on fatty acid oxidation. The potential effects of inhibiting CPTI activity on gene expressions of CPTI and ACO induced by PPARα activation were evaluated via administration of etomoxir, an irreversible inhibitor of the CPTI enzyme located on the outer face of the inner mitochondrial membrane. The effects of malonyl-CoA on the fatty acid oxidation regulation were also investigated via examining *ACC* and *MCD* gene expression. A practical consideration of this research is that it would allow the agonist to be directed specifically to high-risk piglets at risk of energy deficiency. Therefore, the effect of clofibrate on gene expression and activity of lipid-metabolizing enzymes was determined in the liver of pigs at different postnatal ages.

## RESULTS

### Growth performance (body weight, weight gain and liver weight)

Supplementation of clofibrate (Clo) or clofibrate with etomoxir (Clo+Eto) had no effects on initial body weight (P = 0.87), end body weight (P=0.87), body weight gain (P = 0.88), daily gain (P = 0.99), and the ratio of gain/milk consumed (P = 0.88), nor were any differences detected in quantitative image analysis of the liver histology sections (data not shown). The average initial body weight, end body weight, body weight gain, daily gain and ratio of gain/milk consumed were on average 1432.7 g, 1848.9 g, 366.1 g, 70.8 g and 0.05, separately. The average weight gain (g) from d 1 (16.6) to d 7 (812.9) and milk intake (g) from d 1(155) to d 7 (748.9) increased with increasing of postnatal age (P < 0.004). There were no effects of Clo or Clo+Eto on liver weight (P = 0.12). Liver weights (from 36 to 40 g) also increased with increasing body weights (P < 0.004), but relative weight (% of body weight) did not change (P = 0.42) during the experiment. No interactions between treatment and piglet age were detected (P = 0.24).

### Plasma ketone body, free fatty acid and insulin concentrations

**Figure 1:**
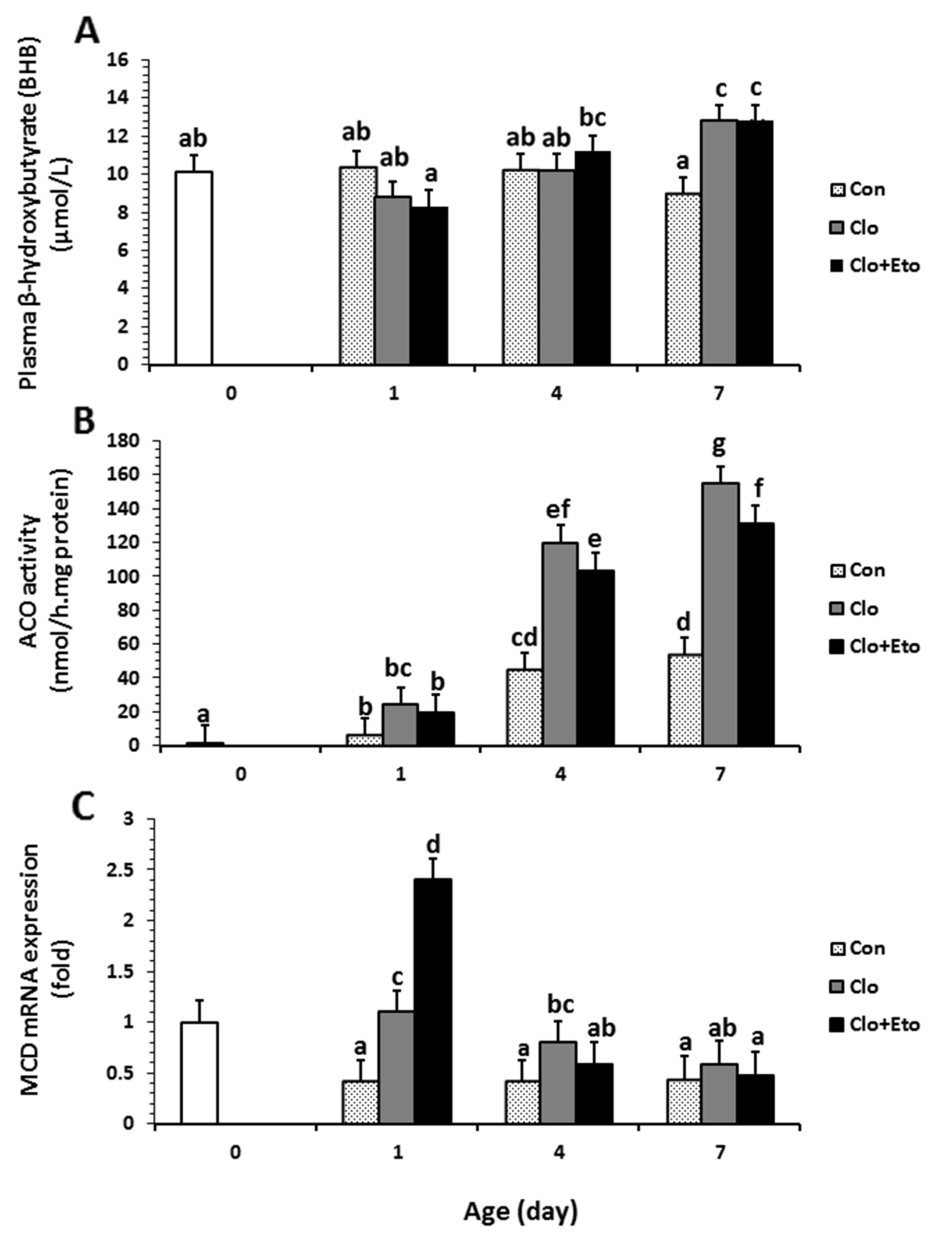
The effects of postnatal age and clofibrate administration on plasma BHB **(A)** and hepatic ACO activity **(B)** and MCD gene expression **(C)**. Values obtained from pigs received vehicle (Con), clofibrate (Clo) or clofibrate + etomoxir (Clo+Eto) at age of d 0 (n=6), 1 (n=18), 4 (n=18) and 7 (n=18). For all panels, treatment^*^age interaction (P ≤ 0.01); values are least square means ± SEM. Bars lacking a common letter differ (P < 0.005).

The average concentration of acetoacetate (AcAc) in plasma of pigs after d 1 was 2.3 fold higher than that at birth, but there were no differences among d 1, 4 and 7. The treatment effect on AcAc concentration and interaction of the treatment with age were not detected (Table 1). Similarly, the average concentration of total ketone bodies (KB = β-hydroxybutyrate (BHB) + AcAc) should be increased 66% after day 1, but no treatment effect or interaction were detected (Table 1). Age and treatment had no effect on plasma insulin concentration. The plasma concentration measured in all pigs on average was 0.035 μg/L (Table 1). Plasma total free fatty acid (TFFA) increased with advancing postnatal age. The concentration (mmol/L) of TFFA was 45% higher in pigs on d 7 than d 4 and 100% higher than that on d 0 and 1. Neither Clo nor Clo+Eto had an impact on plasma TFFA, and no interactions with age were observed (Table 1). Plasma BHB was impacted by postnatal age from d 1 to 7 (P < 0.031). The average concentration (μmol/L) from all treatments determined at d 7 was 19% greater than that determined at birth, d 1 and d 4 (Figure 1A). There was no detectable effect of treatment on BHB, but the interaction of age with treatment was significant (P < 0.013). Treatments with Clo or Clo+Eto increased plasma BHB by 38% over Con by d7 (Figure 1A).

**Table 1:** Plasma ketone body, total free fatty acid and insulin concentrations from pigs that received vehicle (Con), clofibrate (Clo) or clofibrate + etomoxir (Clo+Eto) during postnatal period^*^

	Age (day)				SEM	P-value	Treatments			SEM	P-value	Age^*^treatment
	0	1	4	7			Con	Clo	Clo+Eto			P-value
**AcAc** *(μmol/L)*	9.60^a^	22.92^b^	21.35^b^	23.42^b^	1.11	0.0001	19.31	17.81	20.85	0.97	0.097	0.48
**KB** *(μmol/L)*	19.73^a^	31.09^b^	31.23^b^	34.95^b^	1.36	0.031	28.73	28.30	31.47	0.13	0.13	0.33
**TFFA** *(mmol/L)*	0.044^a^	0.039^a^	0.053^a^	0.077^b^	0.007	0.0014	0.061	0.059	0.048	0.007	0.36	0.74
**Insulin** *(μg/L)*	0.035	0.033	0.036	0.036	0.002	0.52	0.035	0.035	0.035	0.001	0.91	0.29

^*^There was no interaction between age and treatment. The main effects of age (day) and treatment (Con, Clo and Clo+Eto) were presented in the table. Values are means ± SEM for 6 blocks in each age / treatment group. Means without a common letter differ, P < 0.05. AcAc, acetoacetate; KB, total ketone bodies (KB= β-hydroxybutyrate + AcAc) and TFFA, total free fatty acid.

### Enzyme activities

Hepatic CPTI specific activity (Table 2) increased after 24 hours. The activities (μmol/(h·mg protein)) measured on d 1, 4 and 7 were on average 1.9 fold greater than the activity measured in newborns. Administration of Clo stimulated CPTI activity by 1.65 fold of that in Con pigs. Administration of Clo+Eto attenuated the Clo stimulation to Con levels. The sensitivity of CPTI to malonyl-CoA inhibition was impacted by age and Clo (Supplementary Figure 1). The malonyl-CoA inhibition constants (IC_50_) were on average 62% higher at d 7 than at d 1 and 4, and 37% greater from Clo treated pigs than Con pigs (Table 2). Feeding Clo+Eto to piglets had no effect on the IC_50_. The IC_50_ value measured in newborn pigs was 84% greater than that measured in Con pigs on d 1 and 4, but there was no difference between newborns and 7-d-old pigs. No interaction between age and treatment was detected for CPTI activity or IC_50_ values (Table 2).

**Table 2:** Hepatic CPT I and mHMGCS activities from pigs that received vehicle (Con), clofibrate (Clo) or clofibrate + etomoxir (Clo+Eto) during postnatal period^*^

Enzymes	Age (day)				SEM	P-value	Treatments			SEM	P-value	Age^*^treatment
	0	1	4	7			Con	Clo	Clo+Eto			P-Value
*μmol/h.mg protein*												
**CPTI**	30.62	73.65^a^	100.33^b^	89.35^b^	5.01	0.0044	71.08^a^	117.08^b^	75.16^a^	5.03	0.0001	0.95
*μmol/L*												
**IC_50_**	2.39	1.43^a^	1.70^a^	2.53^b^	0.17	0.0004	1.25^a^	2.23^b^	2.18^b^	0.18	0.001	0.25
*nmol/m.mg protein*												
**mHMGCS**	16.25	33.32	47.00	42.46	4.36	0.094	34.84^a^	37.89^a^	50.06^b^	6.11	0.046	0.63

^*^There was no interaction between age and treatment. The main effects of age (day) and treatment (Con, Clo and Clo+Eto) were presented in the table. Values are means ± SEM for 6 animals in each treatment group. Means without a common letter differ, P < 0.05.

CPTI, carnitine palmitoyltransferase I; IC_50_, the half maximal inhibitory concentration of malonyl-CoA (the physiological inhibitor of CPTI) and mHMGCS, mitochondrial 3-Hydroxy-3-methylglutaryl CoA synthase.

Hepatic ACO specific activity was impacted by both age and treatment (Figure 1B). The activity increased with postnatal age regardless of treatment groups (Figure 1B). The interaction between treatment and age was also significant (P < 0.001). In Con pigs, the activity (μmol/mg protein) determined in newborns was increased by 3.2 and 31.2 fold on d 1 and 4. However, the activity measured on d 7 was not different from that measured on d 4 (P = 0.23). Clofibrate stimulated ACO activity and the stimulation was 4.1 and 2.7 fold higher in Clo treated pigs than in Con pigs at d 1 and 4. There was no further stimulation after 4 days. Administration of Clo+Eto reduced the Clo stimulation by 15-20% at 1, 4 and 7 days of age, but the increase in activity with age was of the same order as the piglets treated with Clo only (Figure 1B).

Hepatic mHMGCS specific activities measured on d 1, 4 and 7 were 2, 2.9, and 2.6 times greater than that measured in newborns (P < 0.0001). The activity tended to increase with postnatal age and was 42% higher in 7-d-old than 1-d-old pigs (P = 0.09). Administration of Clo had no effect on the enzyme activity, but addition of Clo+Eto increased the enzyme activity by 44% as compared to Con. No interaction between age and treatment was observed (Table 2).

### Effect of Clo and Clo+Eto administration on peroxisomal and mitochondrial fatty acid oxidation

**Figure 2:**
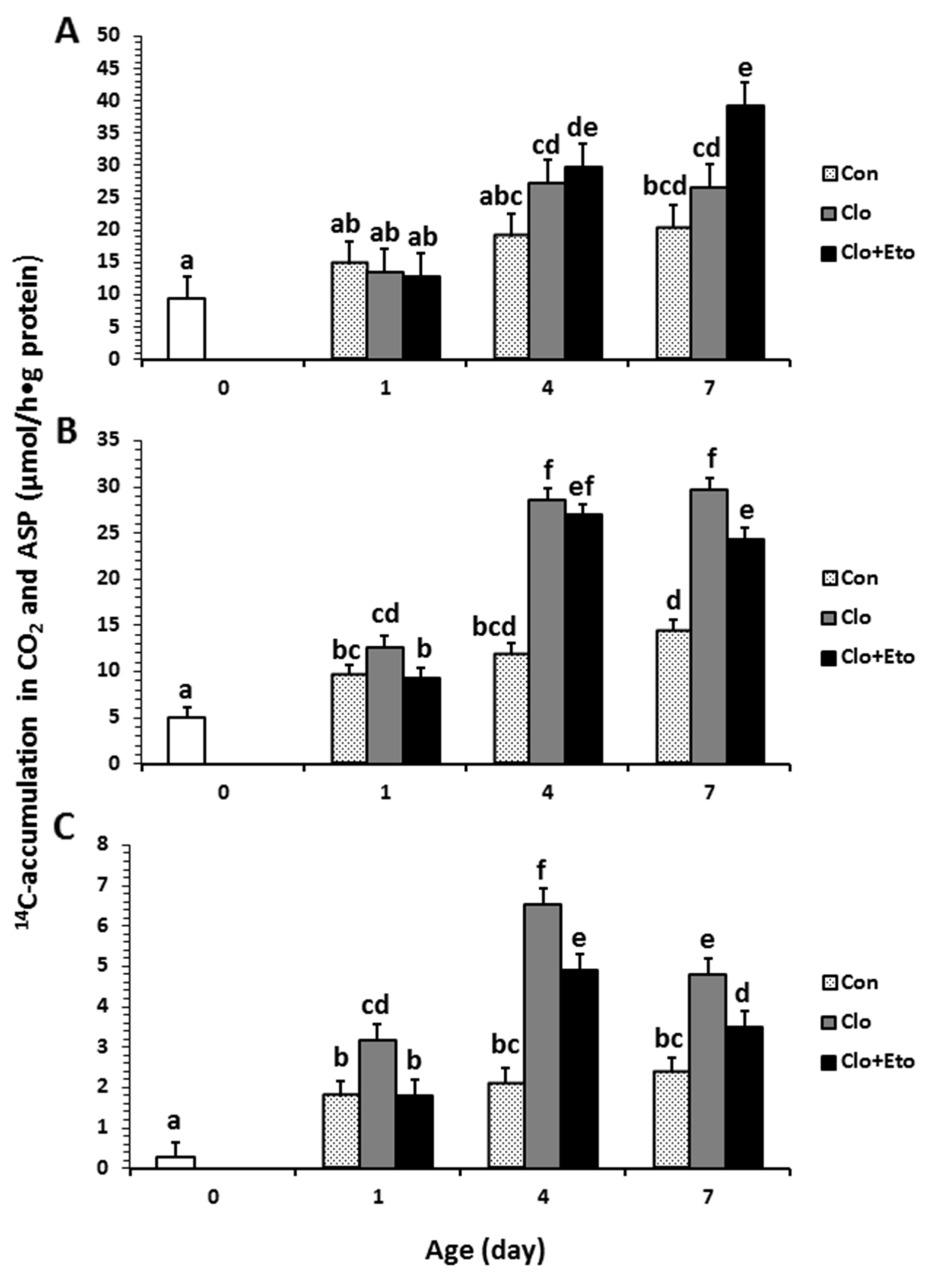
Hepatic peroxisomal fatty acid β-oxidation measured in pigs received vehicle (Con), clofibrate (Clo) or clofibrate + etomoxir (Clo+Eto) The measurements were conducted in fresh liver homogenates isolated from pigs (n=6) at d 0, 1, 4 and 7 using C8:0 **(A)**, C18:1 **(B)** and C22:1 **(C)** as substrate. Values are the least square means ± SEM. Bars lacking a common letter differ (P < 0.005).

**Figure 3:**
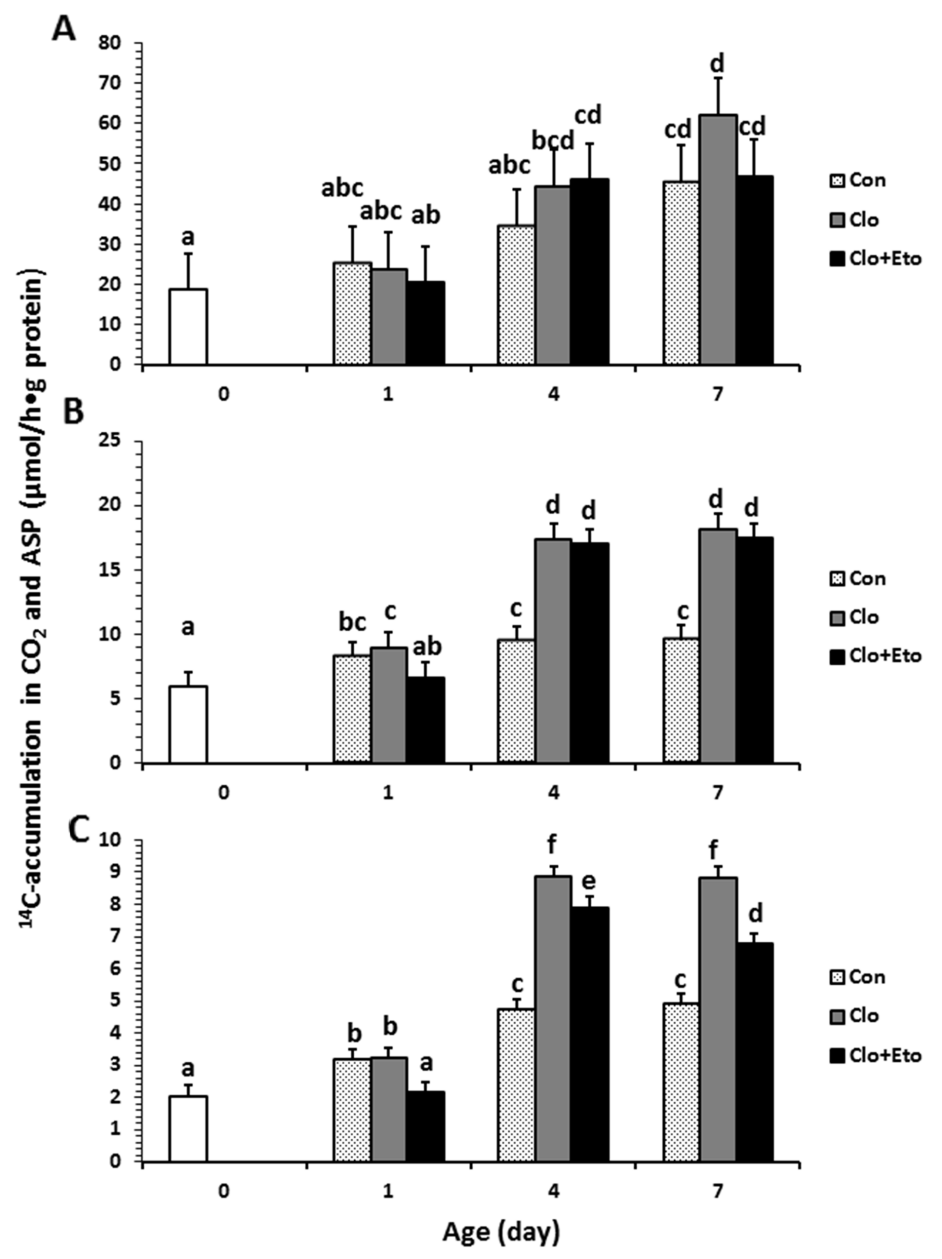
Hepatic mitochondrial fatty acid β-oxidation measured in pigs received vehicle (Con), clofibrate (Clo) or clofibrate + etomoxir (Clo+Eto) The measurements were conducted in fresh liver homogenates isolated from pigs (n=6) at d 0, 1, 4 and 7 using C8:0 **(A)**, C18:1 **(B)** and C22:1 **(C)** as substrate. Values are the least square means ± SEM. Bars lacking a common letter differ (P < 0.005).

**Figure 4:**
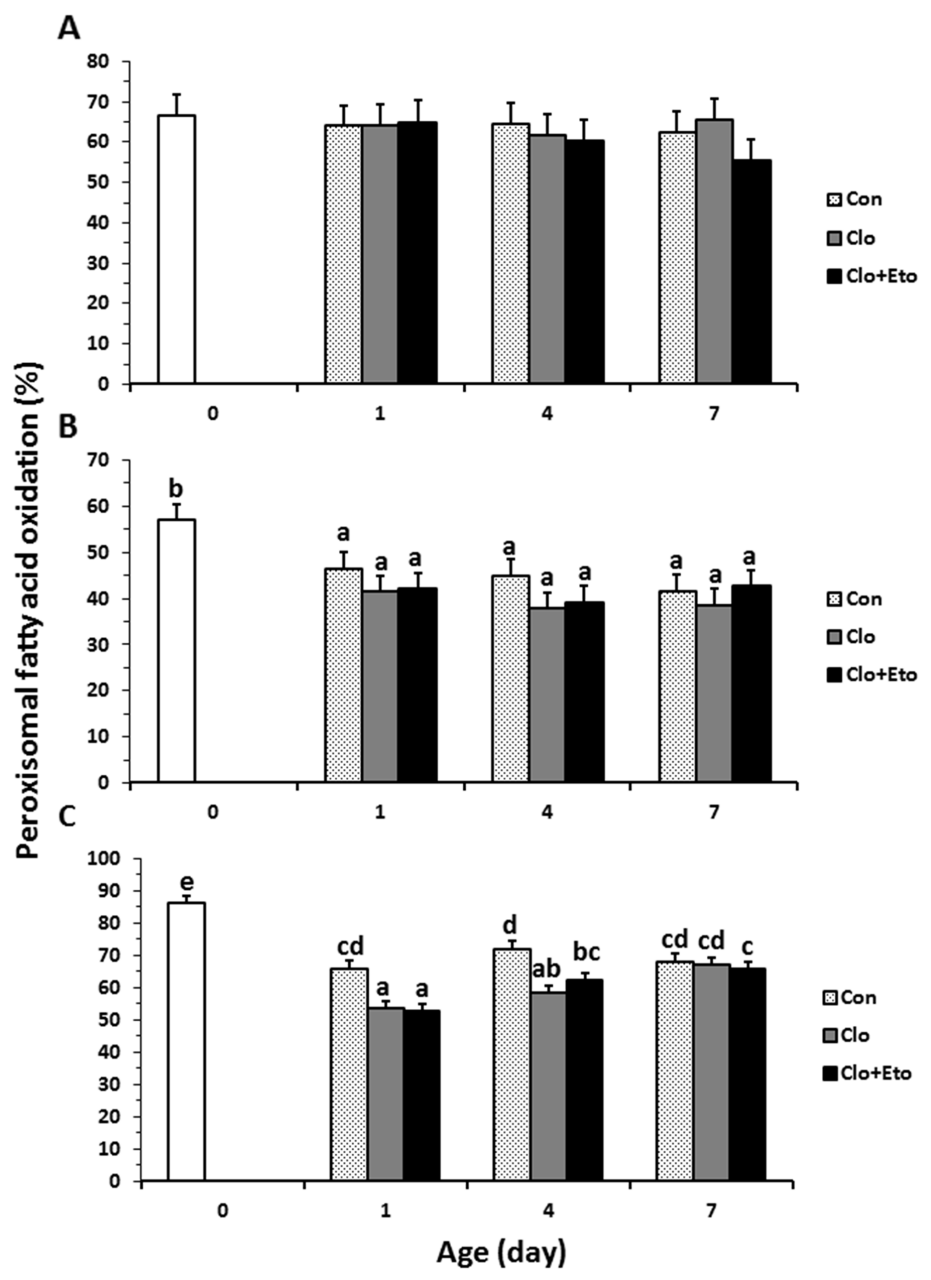
The contributions of peroxisomal β-oxidation to total fatty acid β-oxidation in liver from pigs received vehicle (Con), clofibrate (Clo) or clofibrate + etomoxir (Clo+Eto) The percentage was calculated as the accumulation of ^14^C (CO2 + ASP) in peroxisomes/the accumulation of ^14^C (CO2 + ASP) in both peroxisomes and mitochondria from pigs (n=6) at d 0, 1, 4 and 7 using C8:0 **(A)**, C18:1 **(B)** and C22:1 **(C)** as substrate. Values are the least square means ± SEM. Bars lacking a common letter differ (P < 0.005).

Administration of Clo or Clo+Eto impacted hepatic peroxisomal and mitochondrial oxidation of C8:0, C18:1 and C22:1 CO_2_ and ASP, whereas oxidation rates also differed among the fatty acids (Figures 2–4).

Peroxisomal oxidation rate of C8:0 (Figure 2A) was 20% higher in Clo treated pigs than Con pigs (P < 0.04). Feeding Clo+Eto attenuated the stimulation by Clo, and the oxidation rate (μmol/h·g protein) measured at birth (18.7) was similar to that measured on d 1 (23.2), but was increased by 1.2 and 1.8 fold on d 4 and 7 (P < 0.0005). No interaction between postnatal age and treatment was observed. In contrast, the Clo induction of mitochondrial C8:0 oxidation (Figure 3A) was age dependent (P < 0.017). The mitochondrial oxidation rate was 44% greater in Clo treated pigs than Con pigs on d 4, but no differences between the two groups were detected on d 1 and 7. Feeding Clo+Eto further increased the oxidation rate and the increase was greater on d 7 (94%) than d 4 (57%). Although Clo+Eto influenced both peroxisomal and mitochondrial oxidation, there was no impact on the flux distribution between CO_2_ and ASP. Neither treatment nor postnatal age had an influence on percentage of peroxisomal oxidation (Figure 4A).

Peroxisomal oxidation rate of C18:1 (Figure 2B) was increased on average by 48% in Clo treated pigs as compared to Con pigs (P < 0.0001). Feeding Clo+Eto had no effect on the oxidation induced by Clo. With increasing postnatal age, the oxidation rate measured at birth (5.95 umol/(h·g protein)) increased 34 and 147% on d 1 and 4 (P < 0.0001). There was no further increase after d 4. Similarly, the oxidation rate of C18:1 in mitochondria (Figure 3B) was 85% greater in Clo treated pigs than Con pigs (p < 0.0001). Feeding Clo+Eto attenuated the oxidation rate induced by Clo and the induction (59%) was 25% less as compared to Clo treated pigs (P < 0.0035). The rate also increased with postnatal age as in peroxisomes, and was 1.1 and 3.5 fold higher at d 1 and 4 than at birth. After day 4, the oxidation rate was not increased further (P = 0.61). The percentage of peroxisomal oxidation (Figure 4B) tended to decrease with administration of Clo (P < 0.059), but did not differ between Clo and Clo+Eto treated pigs (P = 0.22). The percentage of peroxisomal oxidation decreased greatly after birth (P < 0.0001), but the decrease was much less after d 4 (P = 0.08).

Peroxisomal of C22:1 (Figure 2C) and mitochondrial oxidation (Figure 3C) followed the same pattern as for C18:1 oxidation. The rate was 48% greater in Clo treated pigs than Con pigs (P < 0.0001). Feeding Clo+Eto had no effect on the Clo stimulation. The oxidation rate in mitochondria was also increased by 85% in pigs fed Clo as compared to Con pigs (P < 0.0001), but the increase was reduced by 16% in pigs fed Clo+Eto (P < 0.0035). No interaction between postnatal age and treatment was detected (P =0.08).

Although Clo greatly stimulated peroxisomal and mitochondrial oxidation rates of all fatty acids, the contribution of peroxisomal oxidation to total oxidation remained unchanged (Figure 4C). The contribution of peroxisomes to beta-oxidation of C18:1 and C22:1 was reduced after one day, but no effect of postnatal age was observed on peroxisomal oxidation of C8:0.

### Effect of Clo and Clo+Eto administration on hepatic gene expression during the neonatal period

The relative mRNA abundance of *L-CPTIα, CPTII, mHMGCS, KetoACoA, ACCβ* and *MCD* measured in the piglets were higher at d 4 than d 1, but no difference was observed between d 4 and 7. Postnatal age had no effects on the mRNA abundance of *ACO, PPARα, L-CPTIβ* or *ACCα*. Supplementation of Clo increased the relative abundance of L-CPTIα (2.7 fold), *mHMGCS* (3.9 fold) and *KetoACoA* (2.4 fold) compared to Con, while supplementation of Clo+Eto had no effect on relative transcript abundance of *L-CPTIα* and *mHMGCS* induced by clofibrate. However, addition of Clo+Eto increased the mRNA abundance of *CPTII* and *L-CPTIβ*, and reduced the expression of *KetoACoA* compared to treatment with Clo alone (Table 3).

**Table 3:** Hepatic enrichment of mRNA associated with fatty acid oxidative genes in pigs that received vehicle (Con), clofibrate (Clo) or clofibrate + etomoxir (Clo+Eto) during postnatal period^*^

Genes	Age (day)				SEM	P-value	Treatments			SEM	P-value	Age^*^treatment
	0	1	4	7			Con	Clo	Clo+Eto			P-value
***Fold***												
**L-CPTIα**	1	1.14^a^	2.21^b^	1.65^b^	0.28	0.032	0.72^a^	1.97^b^	2.30^b^	0.28	0.0011	0.26
**L-CPTIβ**	1	1.54	1.14	0.91	0.33	0.42	0.55^a^	1.31^ab^	1.74^b^	0.33	0.052	0.15
**CPT-II**	1	4.24^a^	1.93^b^	1.25^b^	0.73	0.020	0.98^a^	3.06^ab^	3.39^b^	0.74	0.054	0.43
**ACO**	1	0.85	0.83	0.59	0.12	0.31	0.56	0.84	0.87	0.13	0.18	0.35
**mHMGCS**	1	17.94^a^	38.22^b^	21.29^ab^	6.10	0.05	9.54^a^	36.95^b^	30.95^b^	6.11	0.009	0.88
**PPARα**	1	0.81	0.78	0.56	0.13	0.42	0.56	0.85	0.73	0.14	0.35	0.34
**KetoACoA**	1	0.69^a^	1.52^b^	0.59^a^	0.14	0.0001	0.55^a^	1.34^b^	0.90^a^	0.14	0.0023	0.12
**ACCα**	1	0.41	0.29	0.27	0.06	0.17	0.26	0.30	0.41	0.06	0.19	0.44
**ACCβ**	1	0.66^b^	0.21^a^	0.19^a^	0.15	0.05	0.49	0.30	0.28	0.15	0.56	0.84

^*^There was no interaction between age and treatment. The main effects of age (day) and treatment (Con, Clo and Clo+Eto) were presented in the table. Values are means ± SEM for 6 animals in each treatment group. Means without a common letter differ, P < 0.05. ACO, acyl-CoA oxidase; GAPDH, glyceraldehyde 3- phosphate dehydrogenase; CPT Iα, hepatic carnitine palmitoyltransferase I; CPT II, hepatic carnitine palmitoyl transferase II; CPTIβ, muscle carnitine palmitoyltransferase I; PPARα, peroxisome proliferators activated receptor α; mHMGCS, mitochondrial 3-Hydroxy-3-methylglutaryl CoA synthase; ACCα, acetyl-CoA carboxylase α; ACCβ, acetyl-CoA carboxylase β and MCD, malonyl-CoA decarboxylase.

Supplementation of Clo increased the relative abundance of *MCD* (2.0 fold) compared to Con, but supplementation of Clo+Eto had no effect on the *MCD* expression induced by Clo. An interaction also was observed between treatment and postnatal age for the mRNA abundance of *MCD* (P < 0.001). The administration of Clo stimulated the mRNA expression on d 1, but not on d 4 and 7. The stimulation was greater in pigs with Clo+Eto than with Clo alone. No differences were detected among other treatments (Figure 1C).

## DISCUSSION

Piglet mortality remains high through the first week of life [28], and perinatal programming of energy metabolism is very dynamic, continuing during the early postnatal period. Thus, both the age of the pig and the duration of Clo administration could be important for maintaining high energy utilization. Gene expression induced by Clo changes markedly in adult rats during 14 days of continues treatment, with the highest stimulation for several fatty acid oxidative genes observed by 7 days [29], which implies that the induction of energy utilization could be influenced by the time and duration of Clo administration. Therefore, this experiment was designed to better define the time-dependent effects of postnatal PPARα activation by Clo administration immediately after birth in the suckling pig model.

Consistent with our previous studies [26, 27, 30], Clo administration significantly increased hepatic fatty acid oxidative capacity. The increase was driven by increased CPTI and ACO activities induced by the administration of Clo. The induction followed the same kinetic pattern as the enzymes during early postnatal development (Supplementary Figure 2). The activity showed a burst around 4 d of age and a steady state phase after d4 based on the rate of fatty oxidation measured in this study (Table 2). The postnatal fatty acid oxidation rate followed the same pattern as the enzyme activities. The stimulation by Clo was independent of postnatal age. A linear relationship was observed between the enzyme activity and fatty acid oxidation in both mitochondria and peroxisomes (Supplementary Figure 3). This result indicates that the increase in fatty acid oxidation during the neonatal period induced by Clo is time dependent. A maximum induction would be reached at 4 d of age. The addition of Eto, the inhibitor of CPTI, to the pigs treated with Clo indeed reduced the enzyme activity and impacted long-chain fatty acid oxidation in mitochondria. Similar inhibitory responses were observed also for ACO activity and fatty acid oxidation in peroxisomes. Moreover, the inhibitory impact was related to postnatal age and fatty acid chain length. Interestingly, the inhibitory effect of Eto on ACO activity has never been reported, but both stimulation and inhibition of Eto on *PPARα* and *ACO* gene expression was observed in rats and mice under different experimental conditions [31, 32]. Compared with rodent species, pigs receiving Clo or Clo with Eto showed no effects on the expression of *PPARα* and *ACO* genes. This difference could point to species differences or could be due to administration of Eto with Clo.

Medium-chain fatty acid (C8:0) oxidation increased linearly with postnatal age. The impact of administration of Clo and Clo+Eto on C8:0 oxidation was associated with postnatal age and was greater in pigs at d 4 and 7 than at d 1. However, the ratio of peroxisomal and mitochondrial oxidation for C8:0 remained unchanged. Inhibition of CPTI activity by addition of Eto did not reduce the C8:0 oxidation in mitochondria, suggesting that C8:0 oxidation occurred without CPTI and thus the stimulation of Clo must be via enzymes within the inner mitochondria and peroxisomes. Indeed, both mitochondrial medium-chain acyl-CoA dehydrogenase (*MCAD*) and peroxisomal keto-acyl-CoA thiolase (*KetoACoA*) have been confirmed to be PPARα target genes in the liver of mice [33]. Though we did not measure the expression of *MCAD* in this study, the mRNA enrichment of *KetoACoA* was increased significantly in pigs treated with Clo.

Administration of Clo increased both C18:1 and C22:1 oxidation in peroxisomes and mitochondria, but the increase was correlated to the chain-length and was relatively greater in mitochondria than peroxisomes. Moreover, the ratio of peroxisomal and mitochondrial oxidation was significantly reduced with postnatal age. Administration of Clo or Clo+Eto had no influence on the ratio of peroxisomal and mitochondrial oxidation. Similar results were observed using the same substrates in our previous *in vivo* study [26]. Treatment with Clo+Eto for 7 d reduced oxidation of C18:1 and C22:1, but the reduction of C18:1 occurred in both mitochondria and peroxisome, while the reduction of C22:1 occurred only in peroxisomes. These data confirmed that long-chain fatty acid can be oxidized in both mitochondria and peroxisomes and the oxidation in mitochondria can be increased by the CPTI. The preference of long-chain fatty acid oxidation in peroxisomes increased with the chain-length. The percentage of C22:1 (63.0) oxidation in peroxisomes was 40% greater than that of C18:1(45.5).

Along with the significant increase in the activities of the key enzymes (CPTI & ACO), the mRNA enrichment of *CPTI* increased with postnatal age and Clo administration, which was similar to our previous observation [26]. In addition to *CPTI*, the mRNA enrichment of *CPTII* was also increased with the postnatal age and administration of Clo. The addition of Eto had no influence on its expression. However, the increases in mRNA enrichment of *ACO* and *PPARα* from pigs receiving Clo were not observed when expressed as fold-change versus pigs at birth (0 age), which was also consistent with results observed in our previous work [26]. Because the gene expression might be sensitive to the age and physiological status, all the pigs used in this study were 12-h fasted pigs. Similar results were observed in piglets born to dams fed Clo during gestation [27], demonstrating that transplacental induction is possible. Thus, the changes in *ACO* and *PPARα* gene expression were associated with age and fasting status. Indeed, the influence of the interaction between postnatal age and Clo administration was observed on ACO activity. Furthermore, the induction of peroxisomal β-oxidation by Clo in pigs was smaller as compared to rodent species [30].

Postnatal age and Clo with no Eto had no effect on the activity of mHMG-CoA despite their large stimulation of its mRNA abundance. Nonetheless, plasma ketone bodies (both KB and AcAc) increased with postnatal age.

Administration of Clo not only increased CPTI activity but also reduced the sensitivity of CPTI to malonyl-CoA inhibition, while the addition of Eto had no influence on the sensitivity reduction caused by Clo. Because the crystal structure of porcine CPTI has not been elucidated, the underlying mechanism could not be drawn from this study. It was reported that the isomers of CPTIα and CPTIβ in pigs were different from human and rodent species, affecting their affinity for carnitine and the sensitivity to mlaonyl-CoA inhibition [34]. Because pig CPTIα behaves as CPTIβ in human and rodent species, we measured the abundance of *CPTI* mRNA using specific primers designed for *CPTIα* and *CPTIβ* (Supplementary Table 1). Both pig *CPTIα* and *CPTIβ* were expressed in the liver. The expression of *CPTIα* increased with age and Clo treatment, while the *CPTIβ* message was unaffected by age, but was increased with supplementations of Clo and Eto. Similar stimulatory effects of Eto on *CPTI* gene expression were observed in primary cultures of rat hepatocytes [35]. Whether the changes in expression of *CPTIβ* were associated with the decrease in sensitivity of CPTI to malonyl-CoA inhibition is not known. However, the finding of both *CPTIα* and *CPTIβ* expression in the liver merits further investigation, as does the observed reduction of CPTI sensitivity to malonyl-CoA inhibition and the mechanism of action of Eto.

There is only limited information regarding MCD and ACCβ in the liver of swine, but several lines of evidence suggest that MCD and ACCβ may play an important role in regulating hepatic fatty acid oxidation in pigs. First, lipogenesis activity in the liver is extremely low in swine [36], and the contribution of lipogenesis to lipid synthesis from liver is minimal for this species compared to rats, rabbits and humans [37], suggesting that the amount of malonyl-CoA produced is extremely low in pig liver. Second, results from our previous data [38] indicated that liver CPTI activity is highly sensitive to inhibition by malonyl-CoA in neonatal piglets, and hepatic fatty acid oxidation is increased significantly when the malonyl-CoA concentration is decreased, suggesting that malonyl-CoA generated mainly by ACCβ might serve as the major signaling molecule for metabolic control of fatty acid β-oxidation. Third, inhibition of MCD increases the malonyl-CoA concentration and reduces fatty acid oxidation in pig heart *in vivo* [39], suggesting that malonyl-CoA concentration can be decreased via degradation of malonyl-CoA by MCD. Although the lipogenesis activity is low, it has been shown that both *ACCα* and *ACCβ* are expressed in this species [40, 41]. To explore the regulatory roles of ACCβ and MCD in fatty acid metabolism via malonyl-CoA, the expressions of both *ACCα* and *ACCβ* were measured in this study and the enrichment of *ACCβ* was significantly reduced with postnatal age. Because ACCα maintains regulation of fatty acid synthesis whereas ACCβ mainly regulates fatty acid oxidation [42], this finding indicated that ACC is indeed involved in the regulation of fatty acid oxidation postnatally. Administration of Clo or Clo with Eto had no influence on either *ACCα* or *ACCβ* expression. Campbell and coworkers [43] reported that *ACCβ* expression is not associated transcriptionally with PPARα, but is controlled by sterol regulatory element-binding protein-1 in the liver. Therefore, the result obtained in pig liver contrast observations in rodents. In support of our hypothesis on the role of MCD in fatty acid oxidation, we also found that the expression of *MCD* mRNA was increased significantly in the liver of the pigs receiving Clo, but the increase was associated with postnatal age. The enzyme MCD degrades malonyl-CoA, the physiological inhibitor of CPTI, thus the increase of fatty acid oxidation during the postnatal period could be associated with a reduction in malonyl-CoA concentration due to the activation of MCD, the reduction of ACCβ, or both.

In conclusion, there are no interactions between postnatal age and PPARα activation and expression of its target genes of *ACO, KetoACoA, CPTI* and *mHMGCS* in liver of neonatal pigs. The increased fatty acid oxidation and expression of PPARα target genes induced by PPARα activation are time-dependent in the liver of neonatal pigs. Postnatal changes in *ACC* and modification of *MCD* by PPARα activation observed in this study demonstrated that malonyl-CoA plays an important role in regulation of fatty acid oxidation even though hepatic fatty acid synthesis is very low in pigs. The gene expressions of *CPTIα* and *CPTIβ* detected in the liver might imply that the sensitivity of CPTI to malonyl-CoA inhibition is associated with the expressions of the two isomers, but further study is needed to confirm this hypothesis.

## MATERIALS AND METHODS

### Animals, treatments and experimental design

Sixty newborn pigs (colostrum-deprived) were obtained from the North Carolina State University Swine Education Unit at 12 to 24 h of age. Pigs (1.43 ± 0.06 kg) from 6 litters containing 10 pigs each were used in this experiment. One pig from each litter was selected randomly as a newborn control (New) for sample collection and the remaining nine pigs from each litter were allocated into three treatments by initial body weight (with 3 littermates per treatment). Pigs either received vehicle (2% Tween 80; control, Con), clofibrate (Clo) or clofibrate plus etomoxir (Clo+Eto) in the vehicle once daily via intragastric gavage for up to 7 days. To characterize the roles of CPTI and ACO activities in the increased fatty acid oxidation induced by PPARα activation, etomoxir was used in the treatment of Clo+Eto. Etomoxir is not only an irreversible inhibitor of CPTI [44], but also stimulates *ACO* expression [31]. The clofibrate daily dosage was 75 mg/kg body weight based on our previous research [26] and the daily etomoxir dosage was 25 mg/kg body weight [45] in 5 mL of the vehicle. All pigs were housed individually in cages in an environmentally controlled facility at 30 °C. Pigs were fed colostrum substitute (Colostrum Plus; La Belle Associates, Inc, Bellingham, WA 98226) for 12 h, and then fed milk replacer (containing spray-dried choice white grease as the lipid source) and formulated to containing 25% fat, 31% crude protein, and 36% lactose. Diet was prepared daily and stored under refrigeration, and fresh milk was offered via gravity flow through a nipple to pigs three times per day. Pigs were fed precisely according to a prescribed plan of intake that we had developed (∼60% ad libitum) to match growth rates of sow-reared pigs. Body weights and milk intakes were recorded daily. All procedures were approved by the North Carolina State University Animal Care and Use Committee.

### Tissue sampling and preparation

Pigs were fasted for 12 h and then euthanized by American Veterinary Medical Association (AVMA)-approved electrocution at 1, 4 and 7 days of age. In addition, 6 newborn pigs (one from each litter) were euthanized immediately at birth to serve as day-0 controls. Blood samples were collected and plasma obtained by centrifugation at 2000 × g for 15 min. Liver was removed and weighed immediately, and portions were placed in ice-cold isolation buffer for analyses of CPTI activity in freshly isolated mitochondria and of fatty acid β-oxidation in freshly prepared homogenate. The mitochondria isolation and homogenate preparation procedures were performed as described by Natarajan et al. (2006) [46]. Mitochondrial and homogenate protein concentrations were determined using the Biuret method [47]. Additional liver samples were formalin-fixed for histology examination as described by Cheon et al. (2005) [22]. Liver samples and isolated mitochondrial fractions also were frozen immediately in liquid nitrogen and stored at -80°C for mHMGCS activity and ACO activity and gene expression determinations.

### Plasma analysis

Plasma free fatty acids were analyzed using a kit from BioVision (Catalog# K612-100), and plasma ketone bodies were determined using the procedure as described by Kientsch-Engel and Siess [48]. Plasma insulin was determined using an ELISA kit (Mercodia Inc.; USA).

### Analysis of enzymes

#### CPTI activity and its sensitivity to malonyl-CoA inhibition

CPTI activity in liver mitochondria was determined following the procedure described by Bremer et al. [49] with a slight modification [38].

#### Acyl-CoA oxidase activity

Acyl-CoA oxidase activity was assayed by the fluorometric measurement of H_2_O_2_ using the modified method previously reported by Walusimbi-Kisitu and Harrison (1983) [50]. Reactions with 50μL homogenate containing about 100 μg of protein were protected from light for 10 min at 37°C with shaking. Assays were started by addition of 35 μmol/L palmitoyl-CoA after pre-incubation for 10 min and terminated by adding 4 mL of 0.1 mol/L borate buffer, pH 10 and fluorescence was read at an excitation wavelength of 395 nm and an emission wavelength of 470 nm. Enzyme activity was expressed as nmol H_2_O_2_/(min·mg tissue protein).

### 3-hydroxy-3-methylglutaryl CoA synthase activity

mHMGCS activity was measured at 30°C as described by Quant et al. (1989) [51]. The activity was determined by measuring the disappearance of the enol form of acetoacetyl-CoA by monitoring the absorbance at 303nm for 30 seconds on a spectrophotometer (Beckman DU 600, Fullerton, CA). The absorption coefficient of acetoacetyl-CoA under the conditions used (pH 8.0, 10 mmol/L MgCl_2_), was 12.2 × 10^3^ M^-1^·cm^-1^ [52].

### Analysis of fatty acid β-oxidation

Mitochondrial and peroxisomal β-oxidation were measured in fresh liver homogenates using a modified procedure described previously by Yu et al. [53]. Three fatty acids (sodium salt, 1 mmol/L), [1-^14^C]-caprylic acid (C8:0, 0.2 mCi/mmol), [1-^14^C]-oleic acid (C18:1, 0.2 mCi/mmol) and [1-^14^C]-erucic acid (C22:1, 0.1 mCi/mmol) were bound to fatty acid-free BSA (5:1, molar ratio) and dissolved in the reaction medium. After termination of the reaction, radioactivity in CO_2_ and acid soluble products (ASP) was quantified by liquid scintillation spectrometry (LS-6500 IC; Beckman Instruments, Fullerton, CA). The rate of total β-oxidation was estimated as the rate of accumulation of ^14^C in CO_2_ and ASP. The rate of accumulation of ^14^C into ASP after the addition of the inhibitors (antimycin and rotenone) represented peroxisomal β-oxidation. Mitochondrial β-oxidation was calculated to be the difference between total β-oxidation and peroxisomal β-oxidation [26].

### Relative quantitation of mRNA transcripts

#### Extraction of total RNA

Total RNA was extracted from liver samples using guanidine isothiocynate and phenol (TRI Reagent solution, Sigma-Aldrich, St Louis, MO). Briefly, 50-100 mg of the tissue was homogenized with 1 mL TRI Reagent, and then 0.2 mL of chloroform was added to the homogenate. After centrifugation at 12,000 × g for 15 min, the RNA was precipitated from the aqueous phase by addition 0.5 mL isopropanol followed by centrifugation again at 12,000 × g for 10 min. The pellet was washed with 75 % ethanol and re-suspended in RNase-free water. The RNA then was quantified using a NanoDrop instrument (Thermo Scientific, Wilmington, DE). The integrity of isolated RNA was confirmed using 1 % agarose gel electrophoresis with SYBR Safe™ DNA gel stain (Invitrogen Life Technologies, Grand Island, NY).

#### Reverse transcription

The RNA (10 μg/50 μL) was treated with TurboDNase (Ambion, Austin, TX) according to the manufacturer’s instruction for removal of genomic DNA. Reverse transcription was performed using 2 μg of RNA using iScript™ Select cDNA Synthesis Kit (Bio-Rad Laboratories, Hercules, CA) with random hexamer primers. Final cDNA concentrations were quantified using NanoDrop (Thermo Scientific, Wilmington, DE).

#### Primer design

The primers were purchased from Sigma-Aldrich and Integrated DNA Technologies, and were designed using both PrimerQuest software (Integrated DNA Technologies, Carlsbad, CA) and Primer-BLAST (NCBI). Primer oligonucleotides for *ACO*, glyceraldehyde 3-phosphate dehydrogenase (*GAPDH*), hepatic *CPTIα*, *CPTIβ*, *CPTII*, *mHMGCS*, *PPARα*, *ACCα*, *ACCβ*, and *MCD* were designed with the pig-specific primers except *CPTII* (human; Supplementary Table 1). Primer pairs were selected for optimum annealing temperatures and negligible secondary structure.

#### Real-time RT-PCR

Real-time RT-PCR was conducted using the MyiQ Single Color Real-Time PCR Detection System (Bio-Rad Laboratories, Hercules, CA). All samples were analyzed in triplicate. Optimal primer concentrations for each primer set were determined using a primer matrix before quantification by real-time PCR, and the linearity of amplification for each gene of interest over the 2-log range of cDNA concentrations was verified to be similar to that of the control gene used, *GAPDH*. The control gene was confirmed to be unaffected by clofibrate treatment. Reactions were performed using 12.5 μL of 2 × SYBR Green Supermix (Bio-Rad Laboratories, Hercules, CA) contained 200 ng of cDNA and 0.2 - 0.6 μM of each reverse and forward primers in a final volume of 25 μL. All templates were amplified for 40 cycles under the following conditions: denaturation for 30 s at 95°C, primer annealing and extension for 30 s at 60°C. At the end of amplification cycles, all samples were subjected to melt curve analysis to validate the absence of nonspecific products and primer dimers. Data were collected at the end of each elongation phase. The relative changes in gene expression were determined from the real-time RT-PCR data using the 2^-ΔΔCT^ method [54], where ΔΔC_T_=(C_T,Target_ - C_T.GAPDH_)_time x_ - (C_T.Target_ - C_T.GAPDH_)_time 0_.

### Statistical analyses

Data from growth performance, plasma and tissue enzyme activity and gene expression assays were analyzed using the GLM procedure (SAS) according to a randomized complete block design. The model defined block (litter) treatment group and pig age as independent variables. The Tukey test was used for the multiple comparisons of means. Contrasts were used for the comparison of newborns to other age groups. The treatment and age effects and their interactions were determined according to a 3 (Con, Clo and Clo+Eto treatment) x 3 (1, 4 and 7 d of age) factorial arrangement. When interactions were not statistically significant, only main effects are reported. *in vitro* fatty acid oxidation data were analyzed using a split plot model. The treatment x age effects were assigned to the main plot and the fatty acid effect (C8:0, C18:1 and C22:1) was assigned to the subplot. The differences between the least squares means were assessed with the Tukey test (SAS). All values are presented as least squares means ± SEM, and differences were declared at a p-value of ≤ 0.05.

## SUPPLEMENTARY MATERIALS FIGURES AND TABLE



## Abbreviations

(PPARα): Peroxisome proliferator-activating receptor-α
(ACO): Acyl-CoA oxidase
(CPTI): Carnitine palmitoyltransferase I
(KetoACoA): Peroxisomal keto-acyl-CoA thiolase
(MCD): Malonyl-CoA decarboxylase
(ACCβ): Acetyl-CoA carboxylase β
(mHMGCS): Mitochondrial 3-hydroxy-3-methyl-glutaryl-CoA synthase
(Clo): Clofibrate
(Eto): Etomoxir
(Con): Control
(BHB): β-hydroxybutyrate
(AcAc): Acetoacetate
(BHB+AcAc): KB
(C8:0): Caprylic acid
(C18:1): Oleic acid
(C22:1): Erucic acid
