# Supplementary material for: Pharmacologic activation of peroxisome proliferator-activating receptor-α accelerates hepatic fatty acid oxidation in neonatal pigs
Source: Oncotarget. 2018 May 8;9(35):23900–14. doi: 10.18632/oncotarget.25199 (PMC5963623; doi:10.18632/oncotarget.25199)
Supplement: Supplementary file 1 [file oncotarget-09-23900-s001.pdf]

Pharmacologic activation of peroxisome proliferator-activating receptor-α accelerates hepatic fatty acid oxidation in neonatal pigs

SUPPLEMENTARY MATERIALS

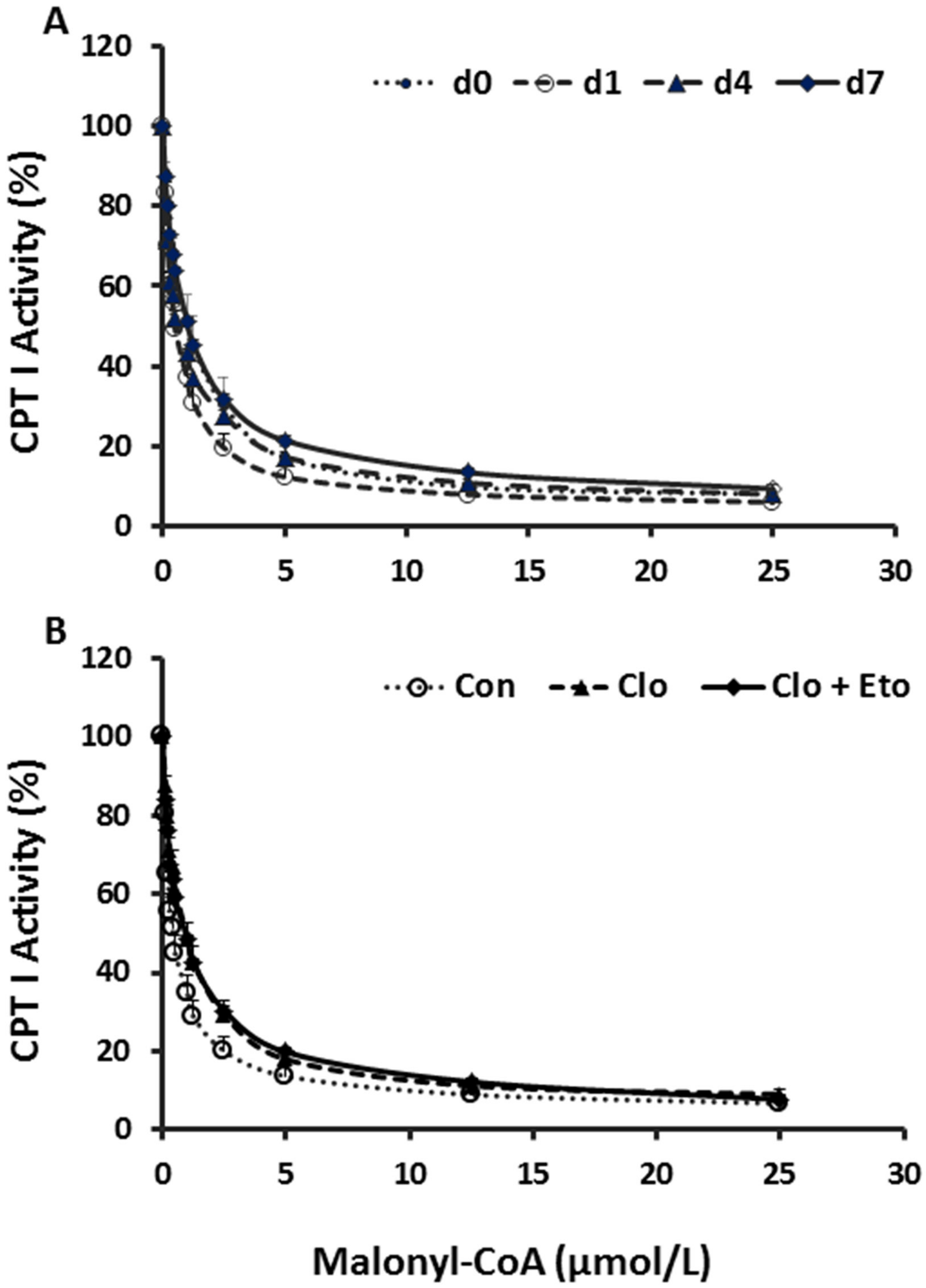

Supplementary Figure 1: The sensitivity of hepatic CPT I to malonyl-CoA inhibition measured in pigs during the postnatal period **(A)** and from pigs received vehicle (Con), clofibrate (Clo) or clofibrate + etomoxir (Clo+Eto) **(B)**. Values in A collected from pigs for postnatal age of d0 (n=6), d1 (n=18), d4 (n=18) and d7 (n=18), and in B obtained from control pig and pigs treated with Clo or clo+Eto (n=18/group). Values are the least square means ± SEM.

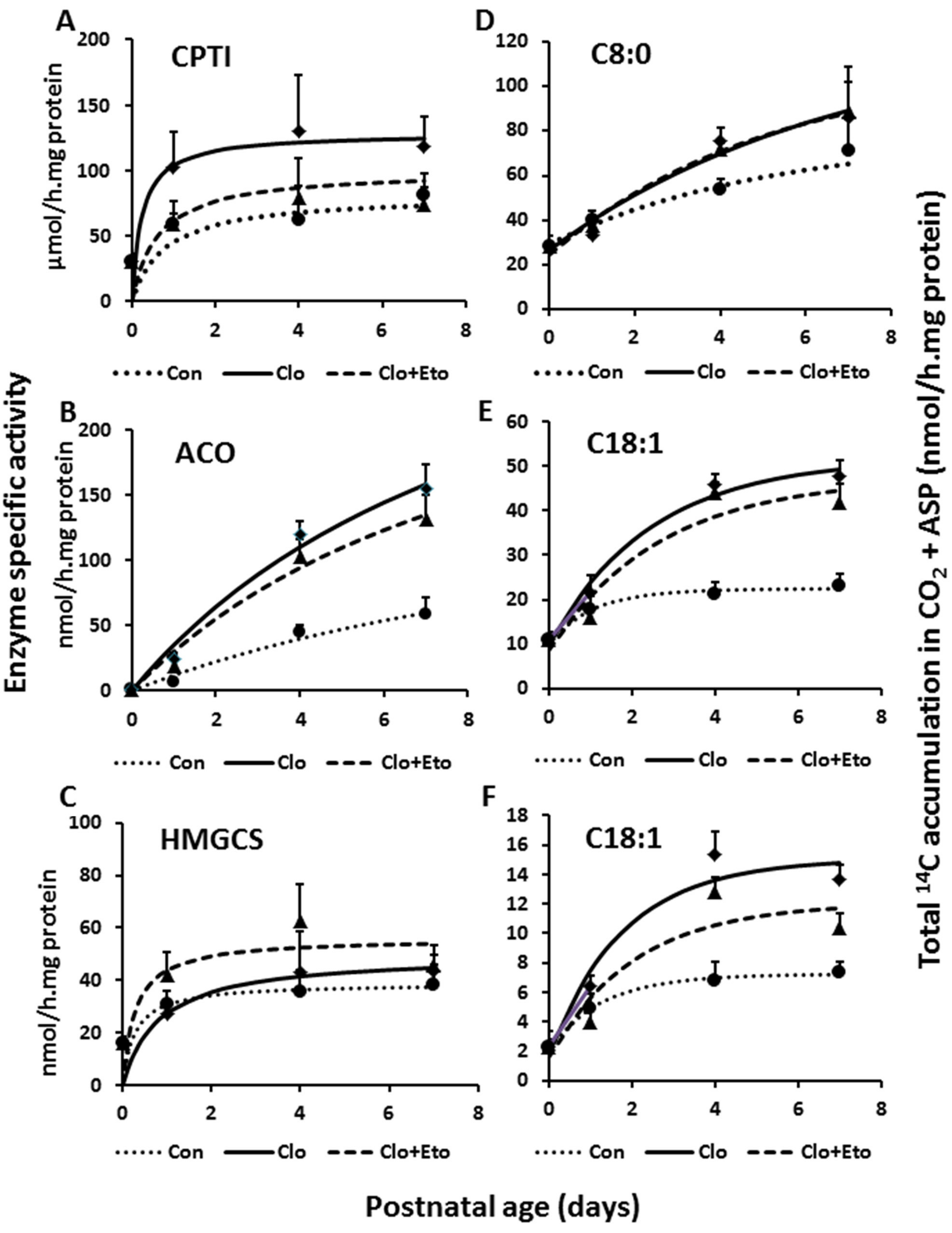

Supplementary Figure 2: Hepatic enzyme activities **(A, B & C)** and fatty acid oxidation **(E, F & D)** in pigs during postnatal period. The effects of postnatal age on enzyme activities and fatty acid oxidation were modeled using the Michaelis-Menten & Hill equations and the values were presented as means ± SEM (n=6 for each age). Con: control, Clo: clofibrate and Clo+Eto: clofibrate plus Etomoxir.

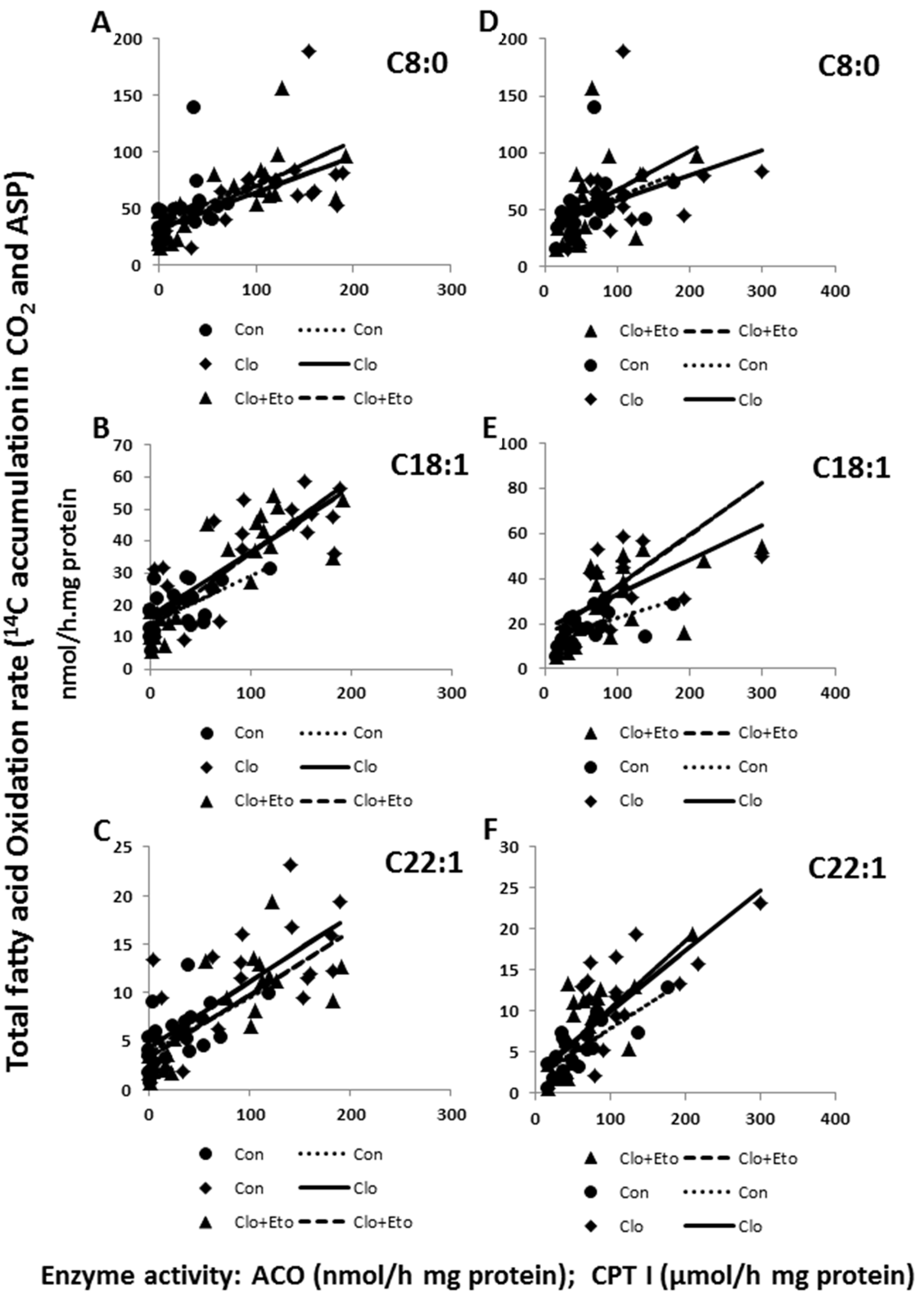

Supplementary Figure 3: Linear relationship between enzyme activities and fatty acid oxidation rate. The regression analyses were conducted using activities of acyl-CoA oxidase (ACO; **A, B & C**) and carnitine palmitoyltransferase I (CPTI; **E, F, & D**) as independent variables (X axis) and oxidation of fatty acids (C8:0: A & E; C18:1: B & F; C22:1: C & D) as dependable variables (Y axis). Con: control, Clo: clofibrate and Clo+Eto: clofibrate plus Etomoxir.

Supplementary Table 1: Characteristics of the specific primers used for real-time RT-PCR

	Forward primer(5'–3'), Sen	Reverse primer(5'–3'), Anti	Amplicon Size, bp	NCBI (Gene Bank)
ACO	CTC GCA GAC CCA GAT GAA AT	TCC AAG CCT CGA AGA TGA GT	218	AF185048
GAPDH	GTC TGG AGA AAC CTG CCA AA	CCC TGT TGC TGT AGC CAA AT	228	AF017079
CPTIα	TCA CAA GCG AAT TTG AGT GC	AAA TTC AGA CCG CAG TTT CG	242	AF288789
CPTII	TGA CCG ACA CTT GTT TGC TC	TGC AGC CTA TCC AGT TGT TG	215	NM_000098; human
CPTIβ	GCA CGC CAG GCC TTC TTC AGC	TGG CCT CGT CTT CCG GGT CAT	121	AY181062
mHMGCS	AAA ACC AAG CCC TCC CTC TA	CCA AGC CAG AGC CAT AAG AG	151	U90884
PPARα	GCC CAA GTT TGA CTT CGC CAT GAA	ATG CAC GAT ACC CTC CTG CAT TCT	151	DQ437887
ACC α	GAA GTC GTC CGG ATC CTC TC	GGC CTC CTA TGC TGT CTC AT	142	NM_001114269
ACC β	AGT CAC CCT TGC TCT TTC ACC TCT	TCA CCA TTG TCC TTG TGG TCC AGT	106	XM_001929270
3-kotoaceyl-CoA thiolase (KetoAcoA)	CAG TTT GAT GTG GTT GTG GC	GCA GGG AGC TCA GGT GAT AG	173	AF028007
MCD	TCT GAG GCT GTG CAT CCC GTT AAA	AGA GAA GAA GTA GCA CCT GCG GTA	81	AK235264

ACO, acyl-CoA oxidase; GAPDH, glyceraldehyde 3- phosphate dehydrogenase; CPT Iα, hepatic carnitine palmitoyltransferase I; CPT II, hepatic carnitine palmitoyl transferase II; CPTIβ, muscle carnitine palmitoyltransferase I; PPARα, peroxisome proliferators activated receptor α; mHMGCS, mitochondrial 3-Hydroxy-3-methylglutaryl CoA synthase; ACCα, acetyl-CoA carboxylase α; ACCβ, acetyl-CoA carboxylase β and MCD, malonyl-CoA decarboxylase.
